# Malaria vaccines since 2000: progress, priorities, products

**DOI:** 10.1038/s41541-020-0196-3

**Published:** 2020-06-09

**Authors:** Patrick E. Duffy, J. Patrick Gorres

**Affiliations:** Laboratory of Malaria Immunology and Vaccinology, National Institute of Allergy and Infectious Diseases, National Institutes of Health, Bethesda, MD USA

**Keywords:** Vaccines, Malaria

## Abstract

Malaria vaccine development entered a new era in 2015 when the pre-erythrocytic *Plasmodium falciparum* candidate RTS,S was favorably reviewed by the European Medicines Agency and subsequently introduced into national pilot implementation programs, marking the first human anti-parasite vaccine to pass regulatory scrutiny. Since the first trials published in 1997, RTS,S has been evaluated in a series of clinical trials culminating in Phase 3 testing, while testing of other pre-erythrocytic candidates (that target sporozoite- or liver-stage parasites), particularly whole sporozoite vaccines, has also increased. Interest in blood-stage candidates (that limit blood-stage parasite growth) subsided after disappointing human efficacy results, although new blood-stage targets and concepts may revive activity in this area. Over the past decade, testing of transmission-blocking vaccines (that kill mosquito/sexual-stage parasites) advanced to field trials and the first generation of placental malaria vaccines (that clear placenta-sequestering parasites) entered the clinic. Novel antigen discovery, human monoclonal antibodies, structural vaccinology, and improved platforms promise to expand on RTS,S and improve existing vaccine candidates.

## Introduction

The malaria vaccine RTS,S/AS01E (brand name Mosquirix^TM^) received a favorable opinion from the European Medicines Agency (EMA) in 2015 after review of its safety and efficacy to reduce clinical *Plasmodium falciparum* malaria episodes in young African children. This was a milestone in vaccine development as the first human parasite vaccine passed the highest level of regulatory scrutiny (referred to as WHO-listed authority maturity level 4 (WLA ML4))^1^. RTS,S/AS01E pilot implementation programs requested by WHO were launched in 2019 to assess safety and benefits during delivery through standard public health mechanisms. Meanwhile, novel malaria vaccine candidate clinical development has continued apace. Some new vaccine candidates seek to improve on the efficacy of RTS,S/AS01E to prevent clinical malaria in African children, while other candidates in the clinic will pursue different indications such as to protect pregnant women from malaria, or to interrupt the parasite’s cycle of transmission and thereby contribute to regional elimination of malaria by blocking *P. falciparum* infection or transmission to mosquitoes.

Over the past 20 years, the rate of new malaria vaccine trials registered at ClinicalTrials.gov, a major venue to register clinical trials that launched in 2000 (https://clinicaltrials.gov/ct2/about-site/history), has remained steady at ~10 trials each year (Table 1). However, trial registrations reflect shifting priorities over time: RTS,S studies maintained a consistent pace throughout albeit with larger sample sizes, while trials that assess whole sporozoite vaccines (WSV) for their safety and efficacy to reduce *P. falciparum* infection episodes increased in frequency in the last decade, as have transmission-blocking vaccines (TBV) that target parasite sexual stages to prevent parasite transmission to mosquitoes. Further, the first vaccine candidates to protect women from placental malaria entered the clinic in the past 5 years, and trials of blood-stage vaccines (BSV) (which target blood-stage merozoites, with the potential to control blood-stage multiplication, or abort infection during the blood stage) decreased in frequency from 2001–2010 to 2011–2020. Interest has increased in the use of vaccines for malaria elimination, or a so-called vaccine to interrupt malaria transmission (VIMT), that could include antigens expressed during pre-erythrocytic, blood-stage and/or mosquito-sexual stage development in order to reduce or halt the spread of parasites in the community^2^. *P. vivax* vaccine trials were registered sporadically, reflecting the dearth of resources dedicated to this neglected disease that afflicts millions each year. Notably, some promising *P. vivax* candidates induced functional activity in Phase 1 trials.

**Table 1:** Malaria vaccine clinical trials registered at ClinicalTrials.gov since 2000.

	Vaccine type	2001–2005	2006–2010	2011–2015	2016–present	Total	Completed
Total		25	53	55	49	182	145
*P. falciparum*	PEV	16	28	42	37	123	99
	RTS,S	7	9	7	8	31	25
	WSV	0	2	17	18	37	29
	BSV	7	24	5	3	39	31
	PMV	0	0	0	2	2	2
	TBV	1*	0	5	5	11	7
*P. vivax*	PEV	1	1	2	0	4	4
	WSV	0	0	1	0	1	1
	BSV	0	0	1	2	3	1
	TBV	1*	0	0	0	1	1

**Fig. 1:**
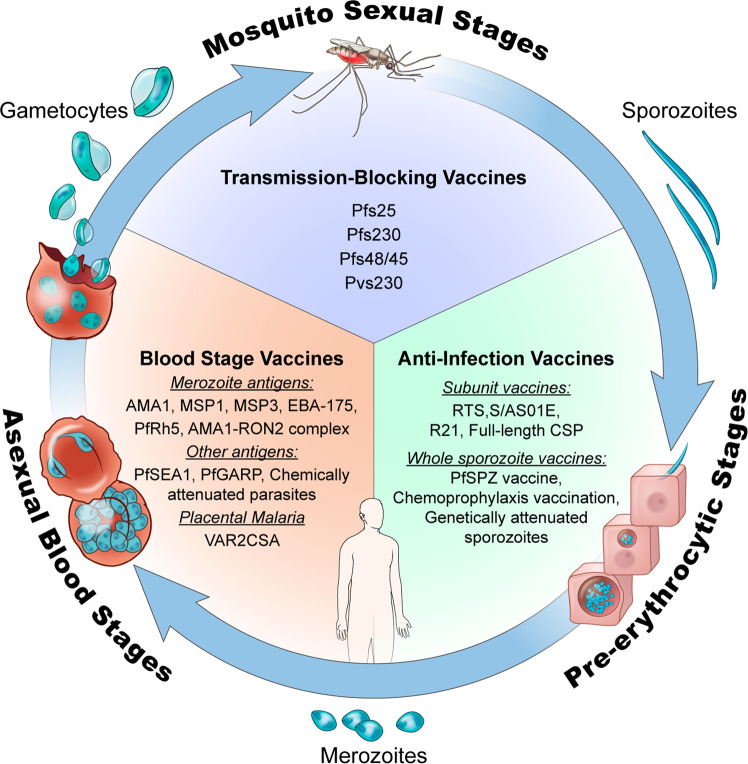
Life cycle stages of *Plasmodium* and vaccine candidates that target each stage. This figure was adapted from a previously published illustration^105^ that has been updated to include more recent malaria vaccine candidates. Illustration by Alan Hoofring, Medical Arts Design Section, NIH.

In this Perspective, we examine the background and rationale for different malaria vaccine concepts that target pre-erythrocytic, blood, or mosquito stages of the parasite life cycle (Fig. 1), we highlight the progress and limitations of several of the most prominent malaria vaccines in or nearing clinical trials since the year 2000 (Table 2), and we describe approaches being used to improve on the existing candidates.

**Table 2:** Selected malaria vaccine candidates currently under preclinical development or in clinical trials.

Vaccine candidate	Immunogen type	Current status
*Pre-erythrocytic stage (anti-infection)*		
RTS,S	Subunit	Phase 4
R21	Subunit	Phase 1/2
Full-length CSP	Subunit	Phase 1
PfSPZ Vaccine	Whole sporozoite (radiation attenuation)	Phase 2
Chemoprophylaxis vaccination (CVac)	Whole sporozoite (chemical attenuation)	Phase 2
Genetically attenuated parasite (GAP) vaccines	Whole sporozoite (genetic attenuation)	Phase 1
*Blood stage*		
PfRH5	Subunit	Phase 1
AMA1-RON2	Subunit	Preclinical
PfSEA-1	Subunit	Preclinical
PfGARP	Subunit	Preclinical
Chemically attenuated parasite (CAP) vaccines	Whole blood-stage parasite	Phase 1
VAR2CSA (Placental malaria)	Subunit	Phase 1
PvDBP (*Plasmodium vivax*)	Subunit	Phase 1
*Mosquito stage (Transmission-blocking)*		
Pfs25	Subunit	Phase 1
Pfs230	Subunit	Phase 2
Pfs48/45	Subunit	Preclinical
Pvs230 (*Plasmodium vivax*)	Subunit	Preclinical

This table was adapted from a previous publication that has been updated to include more recent vaccine candidates^106^. Pre-erythrocytic, blood-stage and transmission-blocking vaccines are being evaluated in clinical trials (denoted as phases I to IV) or are being tested in rodent or non-human primate models (preclinical status).

## Pre-erythrocytic vaccines

Pre-erythrocytic vaccines (PEV) target antigens from *Plasmodium* sporozoite and liver stages, the clinically silent forms that initiate human infection after a mosquito inoculates sporozoites into skin. PEV are designed to induce (1) antibodies against surface antigens that clear sporozoites from skin or bloodstream or block their invasion of hepatocytes, or (2) T cell responses that attack infected hepatocytes. Protective efficacy of PEV was first demonstrated in a human in the 1970s using radiation-attenuated WSV delivered through hundreds of mosquito bites; the vaccinee was protected from subsequent challenge with homologous (i.e., identical strain)^3^ and heterologous^4^ *P. falciparum* sporozoites (PfSPZ) but not from challenge with homologous blood-stage parasites^3^. PEV with high activity can completely clear pre-erythrocytic parasites before release into the bloodstream, and these have also been referred to as anti-infection vaccines (AIV).

### RTS, S and CSP-based vaccines

The demonstration that WSV induce sterilizing immunity in humans coincided with the development of genetic engineering tools. The first malaria gene to be cloned encodes the major surface antigen of sporozoites called circumsporozoite protein or CSP^5^, which continues to be a major focus of vaccine development. RTS,S, the most advanced PEV, incorporates a *P. falciparum* CSP fragment comprising central repeat (hence “R”) and C-terminal regions (containing T cell epitopes, hence “T”) fused to hepatitis B surface antigen (“S”), or altogether “RTS”. RTS is expressed in yeast that also carry hepatitis B “S” expression cassettes, and thus synthesize S and RTS polypeptides that spontaneously co-assemble into mixed lipoprotein particles (or “RTS,S”) with the CSP fragment on their surface^6^.

RTS,S formulated in GSK’s proprietary AS01 adjuvant completed trials in adults, children, and young infants in sub-Saharan Africa^7^. The phase III trial enrolled 15,459 children at 11 centers in seven African countries, and delivered 3 doses at 1-month intervals to coincide with the Extended Program for Immunization schedule, with a booster dose 18 months after the third dose. Clinical malaria episodes (the primary efficacy endpoint) were reduced by ~36% in young children and ~26% among infants who received four vaccine doses (at 0, 1, 2, and 20 months), with statistically significant efficacy against severe malaria in young children but not infants. Efficacy waned over time, with 68% reduction in the incidence of clinical malaria in the first 6 months^8^. The vaccine prevented an estimated 1774 (95% CI 1387–2186; range across sites 205–6565) clinical malaria episodes per 1000 children that received four vaccine doses and 1363 (95% CI 995–1797) per 1000 children that received 3 doses^8,9^. Although vaccine efficacy tended to have a higher point estimate in lower transmission settings, this difference was not significant, and the highest numbers of cases averted were noted in areas of high malaria incidence.

In 2015, the Committee for Medicinal Products for Human Use (CHMP) of the EMA adopted a positive scientific opinion for use of RTS,S outside the European Union “in areas where malaria is regularly found, for the active immunisation of children aged 6 weeks to 17 months against malaria caused by the *Plasmodium falciparum* parasite, and against hepatitis B”^10^. In 2019, a pilot implementation program of RTS,S was launched in Malawi, Ghana, and Kenya to assess protective benefits and safety during routine use in real-life settings. The program will enroll more than a million children over a period of 3 years in selected areas of medium-to-high malaria burden^11^. Children will be randomized to unvaccinated or vaccinated clusters that are offered 4 doses of RTS,S. While WHO views the program as a pilot introduction into national childhood immunization schedules, the program is registered as a study (clinicaltrials.gov ID NCT03806465), prompting ethical concerns over the absence of informed consent, which was considered by WHO to be “implied” as part of the children’s routine vaccination schedule^12^.

Progress with RTS,S represents a historic milestone, but its partial efficacy leaves room for improvement. RTS,S is administered to children according to their age, in order to coincide with other routine EPI vaccinations. The period of peak vaccine-induced antibody levels (and presumed maximum protection) often does not coincide with the malaria parasite transmission season. An ongoing trial in West Africa is using RTS,S for seasonal vaccination with annual booster doses scheduled to maximize antibody responses during the peak malaria parasite transmission season (ClinicalTrials.gov ID NCT03143218). Other field trials are examining fractional dosing regimens that deliver 1/5th of the full dosage for last vaccine administration, a strategy that has increased efficacy against homologous CHMI as well as antibody somatic hypermutation and avidity in malaria-naïve adult subjects^13^.

Another approach is to improve immunogenicity of CSP-based vaccines. At Oxford’s Jenner Institute, a “next-generation RTS,S-like vaccine” called R21 dispenses with the unfused “S” and generates particles solely comprised of CSP-HBsAg fusion protein^14^. In mice, R21 was immunogenic at low doses using human-use adjuvants, and unlike RTS,S, induced minimal antibody responses to the HBsAg fusion partner. Vaccinated mice displayed sterile protection against challenge with transgenic sporozoites expressing homologous PfCSP, and R21 has advanced to Phase 1/2 testing in Africa (ClinicalTrials.gov IDs NCT02925403; NCT03580824; NCT02925403). Viral-vectored PEV candidates (including those that target CSP) have also been assessed in human trials, particularly as part of heterologous prime-boost approaches. These have induced strong CD8 T cell proliferation among other responses, but thus far have failed to exceed the protective efficacy against sporozoite challenge seen with RTS,S (reviewed in ref. ^15^).

As with many *P. falciparum* antigens, CSP displays high sequence diversity including in the C-terminal region targeted by RTS,S vaccine. In sieving analysis of the Phase 3 trial, RTS,S showed greater protection against parasites that matched its C-terminal sequence^16^, indicating that parasite variation can limit efficacy and that escape variants could spread. Speculatively, CSP-based vaccines that eliminate or reduce responses to variant epitopes could improve overall efficacy. Further, the N terminal region is not included in RTS,S, but is critical to hepatocyte attachment and invasion by sporozoites^17^, and naturally acquired antibodies to an N-terminal peptide are associated with protection from disease in Tanzanian children^18^. Full-length CSP candidates have been developed^19,20^ and some have recently entered the clinic [ClinicalTrials.gov ID NCT03589794].

Structural vaccinology approaches are being applied to design improved CSP-based vaccines by defining epitopes of functional human monoclonal antibodies. Monoclonal antibodies to CSP have been prepared from humans after PEV administration or malaria parasite exposure^21–27^. Most anti-CSP human mAbs react to the repeat region, and a subset of these recognize or cross-react to an epitope at the junction of N-terminal and repeat regions. Antibodies to the C-terminal region have been infrequent: only 4 of 215 monoclonal antibodies derived from PfCSP-specific B cells after whole sporozoite vaccination bound the C terminal region specifically^25^. Although the sieving analysis of the RTS,S trial indicated differential efficacy against parasites with C terminal regions that did or did not match the vaccine, the few C-terminal-specific human mAbs tested failed to show functional activity in vitro or in vivo in mice after passive transfer against parasites carrying the homologous CSP sequence^25^. Functional assessment of additional C-terminal-reactive human mAbs may be useful to understand the differential efficacy of RTS,S.

## Whole sporozoite vaccines

Despite evidence since the 1970s that WSV confer sterilizing immunity against sporozoite challenge of humans, WSV were not pursued as a product owing to the perception that manufacture of irradiated sporozoites was impractical for a vaccine^28^. In 2010, the company Sanaria introduced a platform technology that entails harvesting PfSPZ from the salivary glands of aseptic mosquitoes infected by cultured laboratory parasites, followed by purification, vialing, and cryopreservation in liquid nitrogen vapor phase^29^. PfSPZ are attenuated by different approaches to prepare the vaccine candidate product: radiation attenuation (called PfSPZ Vaccine), chemoattenuation achieved in vivo by concomitant administration of antimalarial drugs (called PfSPZ-CVac for chemoprophylaxis vaccination), or genetic attenuation by deletion of genes required to complete liver-stage development^30^ (called PfSPZ-GA1 for the first genetically attenuated PfSPZ candidate (NCT03163121))^31^. PfSPZ Vaccine has required direct venous inoculation to confer sterile immunity against challenge with sporozoites^32^. The logistical and potential cost challenges to implementing WSV will include (1) liquid nitrogen cold chain, (2) intravenous inoculation, (3) scale-up of manufacture.

The efficacy of WSV has been demonstrated in humans although importantly this efficacy is dose-dependent^32–34^. In malaria-naive adults, the level and duration of protection from homologous or heterologous sporozoite challenge depend on dose and regimen with either PfSPZ Vaccine or PfSPZ-CVac, and these have achieved high levels of sterile homologous immunity^32,33,35–37^. Protection against heterologous CHMI and protection beyond a few months have not yet been studied systematically. In an area of intense malaria transmission in Mali, five administrations of PfSPZ Vaccine (2.7 × 10^5^ PfSPZ dosage) to adult residents reduced the risk of new *P. falciparum* infection by 52% in time-to-event analysis over the 24 weeks after last dose, and reduced the proportion infected across the transmission season by 29%^34^. The time-to-event efficacy achieved appears greater than that reported for RTS,S in adults using AS02 or AS01 adjuvants^38,39^. Additional field efficacy trials of PfSPZ Vaccine with 3-dose regimens have been completed in adults and infants (Supplementary Data Set 1) and await publication. In particular, an efficacy trial in Kenyan infants was completed in August 2018 (clinicaltrials.gov ID NCT02687373), and the results of that trial will allow a comparison to efficacy of RTS,S/AS01 in this key demographic group.

In malaria-naive individuals, PfSPZ-CVac using chloroquine conferred high levels of sterile immunity against homologous sporozoite challenge^40^ that lasted for up to 2 years^41^, but induced sterile heterologous immunity in only a minority of vaccinees^42^. Field trials of PfSPZ-CVac have been completed or are ongoing [Supplementary Data Set 1] but results have not yet been published. PfSPZ-CVac approaches are a valuable translational research model to study human sterile immunity. Development as a viable vaccination strategy will require safe and reliable delivery, such as by coformulation of non-attenuated highly sensitive sporozoites and long-lived chemoprophylactic agents to ensure full chemoattenuation in vivo. GAP vaccines are being tested in malaria-naïve individuals for safety, immunogenicity and protective efficacy (clinicaltrials.gov ID NCT03168854; NCT03163121).

Improved field efficacy of WSV will require new regimens or approaches, and future studies will likely incorporate different *P. falciparum* strains in PfSPZ products to broaden efficacy against heterogeneous parasites that naturally circulate. Immunological analysis of WSV trials may guide approaches to improve field efficacy of PfSPZ and other PEV candidate products. WSV express thousands of malaria antigens and induce a broad immune response including CD4 T cells, CD8 T cells, γδ T cells and antibodies. Among these, the Vδ2 subset of γδ T cells and antibodies to the CSP protein have been associated with protection in human trials^37,43^. In SPZ-vaccinated mice, a subset of γδ T cells are required for the induction of protective CD8+ T cells that mediate killing of intrahepatocytic parasites; however, γδ T cells do not directly mediate protection against sporozoite challenge^43^. These findings are consistent with longstanding evidence in mice and in monkeys that CD8+ T cells play a key role in SPZ-induced sterile immunity^44^.

## Blood-stage vaccines

BSV target the asexual parasite forms that undergo repeated multiplicative cycles in erythrocytes and cause disease and death. Cycle duration varies between malaria parasite species and determines the period between fevers, or periodicity: 1 day for *P. knowlesi*, 2 days for *P. falciparum, P. vivax* and *P. ovale*, and 3 days for *P. malariae*. At the completion of each cycle, the brood of ~1–2 dozen progeny (called merozoites) egress from host erythrocytes and within seconds each merozoite has invaded a new erythrocyte to initiate another round of multiplication (and a subset of invasive merozoites commit to generate the sexual forms that will infect mosquitoes).

Blood-stage parasites are an attractive target because this is the disease-causing stage of development, and also because passive transfer of IgG purified from semi-immune African adults was shown to clear parasitemia from African children 6 decades ago^45,46^ and later in Thai adults^47^. Of note, the studies in Africa included children with malaria who did not receive antimalarial chemotherapy as the standard of care^45,46^. and hence would not now pass ethical scrutiny. In subsequent studies, immunization with whole parasite preparations rich in merozoites protected monkeys from *P. falciparum* infection^48^, focusing attention of vaccine developers on merozoite invasion over the ensuing years.

The challenges to developing anti-merozoite vaccines include (1) the brief time (seconds) when merozoites pass between erythrocytes and are accessible to antibodies, (2) antigenic polymorphism, (3) redundant invasion pathways, and (4) the large number of parasites that need to be targeted compared with the numerical bottlenecks attacked by PEVs and TBVs. Between 2000-2015, over 30 BSV trials registered in ClinicalTrials.gov were completed (Table 1), with the large majority targeting the antigens MSP1 and AMA1 and a handful targeting other antigens like EBA-175 and MSP3. In general, these trials sought to elicit high titer antibody against merozoite surface antigens that would impair parasite invasion, or in the case of MSP3, would mediate antibody-dependent cellular inhibition^49^. Ultimately, the results showed scant evidence of protection against controlled human infection or against naturally occurring infection. In particular, AMA-1 candidates induced high titer antibody that was functional by in vitro assays in two trials but failed to show efficacy against controlled infection with the homologous parasite^50,51^. Among all BSV candidates, only GMZ2 (consisting of conserved domains of GLURP and MSP3) showed statistically significant albeit low (14%) efficacy in a pre-specified analysis against naturally acquired infection^52^.

After these disappointments, attention turned to identifying novel BSV antigens or refining the approach to existing targets. Two vaccine candidates seek to address the issue of redundant invasion pathways: PfRH5 and the AMA1-RON2 complex.

### PfRH5

*P. falciparum* reticulocyte-binding protein homolog 5 (PfRH5) binds the essential red cell receptor basigin and shows limited polymorphism^53^, and entered clinical trials using a viral-vectored prime-boost immunogen^54^. PfRH5 is the first highly conserved merozoite antigen shown to induce broadly neutralizing antibody in preclinical studies^55^. In monkeys, different combinations of PfRH5 viral-vectored and/or adjuvanted protein immunogens conferred protective immunity that controlled parasitemia after challenge with virulent heterologous parasites^56^.

Notably, natural infections induce modest or no antibody against PfRH5^55,57,58^ and PfRH5 studies in monkeys showed good protection against virulent blood-stage parasite challenge but modest or no boosting of vaccine-induced antibody by infection^56^. This may limit the duration of protection conferred by a vaccine. In addition, protection in monkeys required an estimated 200 µg/mL of anti-PfRH5 IgG^56^, a high level to achieve and sustain by vaccination. Efforts to improve RH5 vaccine candidates include presentation in virus-like particles (VLP) and production of a protein vaccine in *Drosophila* (Schneider 2) cells^59,60^.

In addition, scientists at Jenner Institute have generated human mAbs from PfRH5 vaccinees and used these in structural studies to identify epitopes targeted by neutralizing, non-neutralizing and potentiating antibodies (the latter slow merozoite invasion and enhance activity of neutralizing antibodies)^61^. This knowledge will inform the design of improved Rh5 immunogens that focus the antibody response on neutralizing and potentiating epitopes.

### AMA1-RON2

Despite its poor efficacy in previous trials, AMA1 is an essential protein for blood-stage parasite growth. The recognition that AMA1 binds to the rhoptry neck protein RON2 at the merozoite-erythrocyte interface to initiate invasion has revived interest in AMA1 as an immunogen in complex with RON2. When complexed with RON2 peptide, AMA1 antigenicity is altered to generate more potent anti-invasion antibodies than monomeric AMA1 antigen^62^. In monkeys, AMA1-RON2 showed significantly greater protection against heterologous blood-stage challenge versus AMA1 alone, and conferred sterile protection in half the animals^62^. As with Rh5, AMA1 vaccines may be improved by structural studies of antigen-antibody complexes to determine epitopes to include or exclude in re-designed immunogens. Unlike Rh5, AMA1 displays extensive sequence variation, and therefore future studies will need to assess the number of alleles or chimeric sequences that will be required for AMA1-RON2 to confer broadly effective immunity.

## Novel BSV antigens

The search for novel BSV antigens has also moved beyond merozoite targets. Parasite antigens are exported to the surface of infected erythrocytes where they are accessible to antibody for hours. Among these, the variant surface antigen family PfEMP1 is immunodominant, mediates parasite sequestration and hence virulence of *P. falciparum*, and is a target of naturally acquired protective antibody^63^. However its highly polymorphic sequence, large size, and cysteine-rich conformational structure have impeded vaccine development and no trials of PfEMP1-based vaccines have been reported. An exception to this is VAR2CSA, a distinctly structured PfEMP1 family member used by the parasite to sequester in the placenta, as discussed in the next section on placental malaria vaccines (PMV).

Interestingly, a non-PfEMP1-infected erythrocyte surface protein called PfGARP has just been described as the target of protective antibodies^64^. Antibodies to PfGARP induced programmed cell death of intraeythrocytic trophozoites in vitro and naturally acquired PfGARP antibodies were related to control of *P. falciparum* parasitemia and protection from severe malaria. In monkey studies, PfGARP vaccines conferred partial protection against *P. falciparum* challenge.

Parasite egress from erythrocytes has also been identified as a target of protective antibody. A differential screen of sera from children that did or did not control parasite density during infection associated protection to antibody against *P. falciparum* Schizont Egress Antigen 1 (PfSEA-1)^65^. Antibodies bind to intraeythrocyic PfSEA-1 and arrest *P. falciparum* schizont rupture in vitro, and vaccination of mice with recombinant *P. berghei* SEA-1 reduced parasitemia and delayed mortality after challenge with lethal *P. berghei*.

Given the disappointing record of subunit BSV in human trials, scientists at Griffith University in Australia are exploring whole blood-stage parasite vaccines attenuated by incubation with a DNA-binding drug (e.g., Tafuramycin-A). Unlike the PfSPZ-CVac approach (described above) that chemoattenuates parasites in vivo, chemically attenuated blood-stage parasites (CAP) are prepared in vitro before administration. In mice, CAP (but not lysed parasites) induced homologous and heterologous immunity; protection was CD4+ T cell-dependent^66–68^ and persisted after CD8+ T cell depletion^67^. In *Aotus nancymaae* monkeys, a single CAP dose did not delay patent parasitemia after blood-stage parasite challenge but may have delayed drug treatment and induced CD8 T cell responses^69^. In humans, CAP were well-tolerated in malaria-naïve volunteers and induced T cell but not antibody responses^70^. A human trial of a 3-dose CAP regimen has been registered to assess efficacy against challenge with homologous blood-stage parasites [ACTRN12618001314213]. As with PfSPZ-CVac, CAP will need to be convincingly shown to be safe and implementable to be viewed as a viable vaccination strategy.

## Placental malaria vaccines

PMV target chondroitin sulfate A (CSA)-binding parasites that uniquely sequester in the placenta; hence PMV represent a distinct BSV approach. While vaccines such as PEV and BSV candidates that protect the general population may also benefit pregnant women, naturally acquired protection against placental malaria offers a focused vaccine approach. Natural antibodies to CSA-binding parasites are associated with protection from placental malaria and are acquired over successive pregnancies as women in endemic areas become resistant to placental malaria^71^. Placental parasites uniformly express the distinctive PfEMP1 family member VAR2CSA that binds CSA^72^; recombinant VAR2CSA induces antibodies that block parasite binding to CSA (reviewed in ref. ^73^). VAR2CSA is a complex target that has a large (>300 kD) extracellular domain with six DBL domains and additional interdomain regions, and a recent report identified atypical VAR2CSA with seven or eight DBL domains in some field isolates that can be functional^74^.

The first trials of VAR2CSA-based vaccines have been conducted over the past 5 years. Owing to its large size, VAR2CSA vaccine development has focused on individual domains or domain combinations. Two candidates based on N-terminal VAR2CSA fragments that have high binding affinity for CSA have completed first-in-human trials. The *Drosophila* cell-expressed PAMVAC was tested in different human adjuvants and proved to be safe, well-tolerated, and induced functionally active antibodies against homologous parasites^75^. PAMVAC will be tested in malaria-experienced nulligravidae next. A second subunit VAR2CSA candidate, PRIMVAC, has completed a first-in-human trial in France and Burkina Faso, which showed the vaccine was safe, immunogenic, and induced functional antibodies against the homologous VAR2CSA variant expressed by NF54-CSA infected erythrocytes. However, cross-reactivity against heterologous VAR2CSA variants was limited and only observed in the higher dose group^76^. Researchers hypothesized that an alternate schedule of immunization, antigen dose, and combinations with other VAR2CSA-based vaccines could improve the cross-reactivity against heterologous VAR2CSA variants.

## Transmission-blocking vaccines

TBV incorporate surface antigens of mosquito/sexual-stages (gametes and zygotes) in order to induce antibodies that kill parasites in the mosquito bloodmeal and interrupt parasite transmission through the vector^77,78^. Target antigens were identified with monoclonal antibodies that were raised in rodents against gamete/zygote preparations and blocked infection of mosquitoes. The four leading candidates have been grouped as gamete surface proteins first expressed by gametocytes in human blood^79^ such as Pfs230 and Pfs48/45 of *P. falciparum*, and zygote surface proteins expressed only post-fertilization in the mosquito host^80,81^ such as Pfs25 and Pfs28. These antigens are cysteine-rich with multiple 6-cys or epidermal growth factor (EGF)-like domains that have been challenging to prepare as properly folded recombinant protein. Pfs25 was the first TBV candidate prepared as a recombinant protein^82^. In animal studies, Pfs25 candidates have induced equal or greater serum transmission-blocking activity as other antigens or antigen combinations^83,84^ and hence Pfs25 has been the focus of clinical trials published to date. Ongoing trials are now examining the activity of Pfs230 vaccine candidates (ClinicalTrials.gov IDs NCT02942277; NCT03917654). Pfs230 antibodies raised in animals show lytic activity against *P. falciparum* gametes in the presence of complement^85^, which might similarly enhance activity of human Pfs230 antisera.

Both Pfs25 and Pfs230 recombinant antigens have shown poor immunogenicity as monomers. To enhance immunogenicity, our group prepares protein-protein conjugate vaccines by chemically coupling *Pichia-*expressed Pfs25 to carriers such as ExoProtein (EPA) to generate nanoparticles, and formulate these in adjuvants^86^. While several previous trials of Pfs25 candidates failed to induce adequate antibody responses or were overly reactogenic in human vaccinees, Pfs25-EPA conjugate formulated with Alhydrogel® was reported in 2016 to be well-tolerated and to induce functional antibodies in humans that block transmission of *P. falciparum* to mosquitoes in membrane feeding assays^87^, and this activity correlated with titers. However, functional activity in most vaccinees required 4 doses and antibody titers and activity waned rapidly.

Ongoing studies (ClinicalTrials.gov ID NCT02334462) are comparing and combining Pfs25 and Pfs230 vaccine antigens using *Pichia-*expressed Pfs230 domain 1^88^. These studies are also assessing the benefits of alternative adjuvants, including the GSK adjuvant AS01 used in the RTS,S vaccine (ClinicalTrials.gov ID NCT02942277; NCT03917654).

Additional TBV candidates will enter the clinic in the coming years and can be compared or combined with the current candidates. Gamete surface antigen Pfs48/45 is likely to be the next target tested in humans. Like Pfs230, Pfs48/45 expression occurs during the later stages of gametocyte development in the human red cell. Once ingested by mosquitoes, gametocytes egress from red cells as gametes. Pfs48/45 appears as GPI-anchored antigen on both male and female gametes^89^, where it forms a complex with Pfs230^90^. Pfs48/45 and Pfs230 play a role in male gamete fertility^91^. Pfs48/45 comprises three 6-cys domains, of which the C-terminal domain contains a conformational epitope targeted by potent transmission-blocking mAbs^92^.

Pfs48/45 vaccine development has been hindered by difficulty in recombinant expression of properly folded protein. Progress has recently been reported for the C-terminal 6-Cys domain as the downstream partner in a fusion with the R0 region of asexual stage Glutamate Rich Protein by expression in *Lactococcus lactis*^93^. The resulting antigen, called R0.6 C, reacts to conformation-dependent functional monoclonal antibodies and induces transmission-blocking antibodies in animals. Further, a chimeric protein comprising the pro-domain of Pfs230 (upstream of domain 1) and the C-terminal domain of Pfs48/45 induced significantly higher serum functional activity than did R0.6C, suggesting an additive effect of antibody to Pfs230 and Pfs48/45^94^.

Current challenges of TBV development include achieving sufficient adaptive responses that maintain high levels of antibodies over time, as well as widespread coverage to accomplish herd immunity. Furthermore, TBVs must have an exceptional safety profile since they do not confer direct benefit to the individual. TBVs could be implemented in combination with a PEV to prevent both infection in humans and transmission to mosquitoes, and could similarly be combined with BSV that reduce transmission to assess additive or synergistic activity.

## Vivax vaccines

*P. vivax* causes an estimated 14.3 million malaria episodes each year and is the leading cause of malaria in Asia and Latin America^95^. Although it has been historically designated as benign tertian malaria, *P. vivax* is increasingly recognized as a public health threat causing severe morbidity and mortality^96^. Further, sterile heterologous immunity against *P. vivax* has been demonstrated^4,97^. Despite this, *P. vivax* research suffers from a dearth of resources since the funds dedicated to malaria research—which are not commensurate to the scope of the problem in any case—are predominantly allocated to *P. falciparum* research. This inadequate investment is particularly short-sighted, since vaccines may disproportionately benefit *P. vivax* control: dormant liver forms called hypnozoites produced by *P. vivax* (but not by *P. falciparum*) allow the parasite to relapse repeatedly over months or years and thwart efforts to control or eliminate this species, hence the benefit of durable immunological protection conferred by vaccines.

*P. vivax* vaccine development has generally followed *P. falciparum* efforts. Vivax vaccines tested in humans include orthologues (PvCSP and Pvs25, respectively) of the PEV (PfCSP) and TBV (Pfs25) candidates that have commanded greatest attention for *P. falciparum*. However, PvCSP vaccine prepared as a monomer formulated in GSK’s AS01 adjuvant failed to induce sterile protection against challenge with *P. vivax* sporozoites^98^, and Pvs25 expressed in *S. cerevisiae* formulated in Montanide ISA 51 caused systemic reactogenicity that prompted termination of the clinical trial^99^. Notably, when formulated in Alhydrogel®, Pvs25 was well-tolerated, and the antibody responses, though modest, showed functional transmission-blocking activity in mosquito feeding assays that correlated to antibody concentration^100^. Based on clinical progress with the *P. falciparum* candidate Pfs230D1-EPA, the *P. vivax* candidate Pvs230D1-EPA is currently being manufactured in anticipation of trials that may launch in 2021.

*P. vivax* BSV trials have focused on Duffy-Binding Protein (PvDBP) which binds the Duffy Antigen Receptor for Chemokines (DARC) on erythrocytes and is required for merozoite invasion. Two DBP candidates have completed Phase 1 trials, including a viral-vectored^101^ and a recombinant protein candidate^102^. Both candidates induced strain-transcending functional antibodies measured in vitro. Using human mAbs generated through vaccination or natural vivax exposure, structural studies have identified functional and non-functional epitopes that will provide a rational basis to improve the design of PvDBP immunogens^103,104^.

## Conclusions

The landscape for malaria vaccines in 2020 is very different from that in the year 2000. The pre-erythrocytic vaccine (PEV) product RTS,S/AS01E has proven to be safe and efficacious for reducing clinical malaria in African children. Upon completion of ongoing implementation programs in 2022 in three African countries, the results will be reviewed by international bodies including WHO and RTS,S/AS01E will be considered by national policy decision-makers for broader use in Africa.

While RTS,S reduces clinical malaria risk in African children, newer PEV candidates such as R21/Matrix M, PfSPZ whole sporozoite vaccines, and full-length CSP immunogens seek to improve on its efficacy. In parallel, TBV have advanced to Phase 2 clinical trials over the past decade. Efficacious TBV can be combined with the most effective pre-erythrocytic vaccines to pursue malaria elimination programs in combination with other malaria control tools. The substantial progress made with *P. falciparum* vaccine candidates that have demonstrated efficacy or activity in human trials justifies increased investment in *P. vivax* vaccines to pursue similar goals.

BSV that target merozoite invasion proteins have delivered disappointing efficacy results in clinical trials over the past 20 years. Novel or improved immunogens that target non-redundant merozoite invasion pathways may improve on these dismal results. Meanwhile, vaccines against other BSV targets such as infected red cell surface proteins, schizont egress antigens, or intact infected erythrocytes that have been attenuated, are progressing in preclinical and clinical studies.

PMV represent a distinct type of BSV by targeting the surface antigens of CSA-binding infected erythrocytes that sequester in intervillous spaces and cause placental malaria. Two vaccine candidates that target VAR2CSA, the immunodominant surface antigen of CSA-binding infected erythrocytes, have completed first-in-human trials. An initial report suggests that these vaccines can induce functional activity against homologous parasites. Future studies will determine whether they can induce heterologous activity that is boosted during naturally occurring pregnancy malaria infections to confer durable protection over successive pregnancies.

Malaria vaccine candidates are progressing in clinical trials and RTS,S has advanced to implementation. The question remains how well can malaria vaccines work, and how can we best deploy them to the advantage of the communities devastated by malaria. Scientists are pursuing antigen discovery, structural vaccinology studies, and improved platforms to expand on or improve our existing portfolio of candidates. As the portfolio advances in development, adequate resources are needed to develop promising candidates; as more candidates transition to products, we must ensure these valuable new interventions are optimally deployed to maximize their benefits in the fight against this ancient scourge.

## Supplementary information

supplementary-materials

Supplementary Data Set 1

## Data Availability

All relevant data are included in the submitted manuscript.
