# Supplementary material for: Malaria vaccines since 2000: progress, priorities, products
Source: NPJ Vaccines. 2020 Jun 9;5:48. doi: 10.1038/s41541-020-0196-3 (PMC7283239; doi:10.1038/s41541-020-0196-3)
Supplement: Supplementary file 1 — supplementary-materials [file 41541_2020_196_MOESM1_ESM.pdf]

SUPPLEMENTARY MATERIAL

Supplementary Data Set 1. Detailed list of malaria vaccine clinical trials registered at ClinicalTrials.gov since 2000, including study title, status, intervention, enrollment, start date, location, and vaccine target.
